# Enhancing Success of Veterinary Visits for Clients With Disabilities and an Assistance Dog or Companion Animal: A Review

**DOI:** 10.3389/fvets.2019.00044

**Published:** 2019-02-25

**Authors:** Emma K. Grigg, Lynette A. Hart

**Affiliations:** Department of Population Health and Reproduction, School of Veterinary Medicine, University of California, Davis, Davis, CA, United States

**Keywords:** veterinary care, client communication, assistance dog, therapy animal, special needs, disabilities, dementia, service dog

## Abstract

Despite increasing information on enhancing client communication and compliance/adherence in veterinary medicine, literature focusing on special cases remains limited: working with clients with special needs, challenges or disabilities, or when the patient is an assistance or emotional support animal. This paper summarizes current recommendations on how best to build successful working relationships with these clients, including action items to implement in practice. In addition, this paper reviews current literature on important considerations for care of assistance dogs as patients.

## Introduction

Despite increasing information on enhancing client communication and adherence in veterinary medicine, literature focusing on special cases remains limited: working with clients with special needs, challenges or disabilities, or when the patient is an assistance or emotional support animal. Although the body of research measuring benefits of companion and assistance animals for people with disabilities is growing, much of the available literature on working with clients with disabilities is based on expert opinion. Additional research into best practices would be beneficial to ensure that all practices are evidence-based and effective for both the animals and the human clients. This paper summarizes current recommendations on how best to build successful working relationships with these clients, including action items to implement in practice, and important considerations for providing veterinary care to patients working as assistance animals.

## Relevance for Veterinary Practice—the Numbers and Policy

According to the U.S. Census Bureau, about 56.7 million Americans (19% of the population) were living with a disability in 2010, with more than half describing the disability as severe (1). Of these, ~8.1 million Americans had difficulty seeing (including 2 million blind), 7.6 million had trouble hearing (1.1 million with severe difficulty), and 30.6 million Americans had mobility problems (often requiring a cane, walker, or wheelchair). Not all of these disabilities are “visible”: depression and/or anxiety at levels that interfere with normal daily functioning were reported by 7 million adults. Older Americans were more likely to have a disability than younger Americans, with ~2.4 million Americans with Alzheimer's disease, senility or dementia. Almost 1/3 of all US families (~20.3 million) are impacted by disabilities (2). Pet ownership in the US is high, with 68% of all U.S. households including a pet, and numbers have increased over the last two decades (3). It is probable, therefore, that many Americans living with a disability will have pets. In addition, the number of Americans with a disability is increasing; between 2005 and 2010, the total number of people with a disability increased by 2.2 million; the number (and percentage) with a severe disability also increased. Correspondingly, numbers and percentages of people needing assistance also increased (1). Current estimates report there are ~75 million people over the age of 65 in America, and it is estimated that by 2030, ~50% of these may have a disability (2). For these reasons, it is likely that veterinarians (and other practitioners) will see an increase in clients with disabilities in the near future (4). These clients should have their needs met and receive the same high quality of care provided to clients without disabilities. Sensitivity toward and inclusion of clients with disabilities makes good business sense, and can help both build the veterinary practice and better serve clients and patients (5, 6).

These disabilities frequently have significant impacts on the lives of these individuals. For example, Americans with a disability are less likely to be employed: 41% of Americans between the ages of 21–64 with a disability were employed, compared to 79% of Americans in the same age range without a disability; and they tend to earn less: median income of Americans with a disability is <70% of median income for those without a disability (7). Americans with a disability are more at risk of experiencing persistent poverty (defined as continuous poverty over a 2 year period): nearly 11% of Americans between the ages of 15 and 64 with severe disabilities, and nearly 5% of those with a non-severe disability, experienced persistent poverty, compared to 3.8% of Americans with no disability (1). Challenges are faced on a daily basis: 9.4 million non-institutionalized Americans reported having difficulty with at least one typical activity such as bathing, dressing, and eating (1). Health care has been noted as an area that has been “slow to progress” toward equal accessibility for those living with a disability (2). In a 2012 study of visually-impaired persons in the UK, authors documented an “extremely worrying” lack of access to medical facilities such as doctor's surgeries, with 33% of visually-impaired persons reporting difficulty in accessing services, and 36% reporting frequently leaving without having achieved their objectives for the visit. It is unlikely, these authors note, that veterinary practices perform any better than their human medicine counterparts (4).

In the US, the Americans with Disabilities Act (ADA), originally established in 1990 and revised in 2008, is the primary law designed to ensure that people with disabilities have the same rights and opportunities as people without disabilities. The ADA prohibits discrimination and ensures equal or reasonable accommodation for persons with disabilities in employment, state and local government offices, public accommodations (including private businesses providing goods or services to the public), commercial facilities, and transportation (2). To be considered as having a disability under the ADA, a person must satisfy at least one of the following requirements: (1) have a physical or mental impairment which substantially limits one or more of a person's major life activities, such as walking, seeing, sitting, breathing, etc.; (2) have a record of such impairment; (3) be regarded by the covered entity as having such an impairment (with the covered entity consisting of any organization subject to ADA rules against discrimination) (2). The ADA requires most businesses and facilities to provide reasonable access and accommodation for all disabled customers, clients, and members of the public; this applies to almost all businesses that are open to the public, regardless of size. Reasonable accommodation refers to “necessary and appropriate modification and adjustments not imposing a disproportionate or undue burden, where needed in a particular case, to ensure to persons with disabilities the enjoyment or exercise on an equal basis with others of all human rights and fundamental freedoms” (8). Veterinarians could be impacted by the ADA with regard to how they treat animals owned by clients with disabilities, how they hire those with disabilities, and how facilities are designed and operated (9–11).

An increasing number of studies have documented that individuals with disabilities can benefit from interactions and/or partnership with non-human animals. For instance, pets provide benefits for individuals living with mental health issues [reviewed in Brooks et al. (12)], such as reduced feelings of loneliness and depression in military veterans with post-traumatic stress disorder (PTSD) (13) and increased social interactions and connectivity in patients living with a long-term mental health condition (14). More structured animal-assisted interventions (AAI) show benefits for human participants [reviewed in Bernabei et al. (15) and Charry-Sánchez et al. (16)], such as significant decreases in aggressiveness, anxiety, and caregiving burden in elderly patients with Alzheimer's and dementia following six bi-weekly AAI sessions (17), and reductions in loneliness scores for patients with clinical depression who participated in one or more animal-assisted therapy (AAT) sessions over the course of 6 weeks (18). Assistance dogs have been reported to provide significant social and logistical support for persons with disabilities [reviewed in Winkle et al. (19)], such as increased social greetings and approaches when with a service dog (20, 21), as well as decreased need for paid assistance, and increased self-esteem and feelings of well-being (9, 19, 22–27).

The exact numbers of assistance animals working in the US is unknown, but available data suggest that this number is substantial and increasing (27). Domestic dogs are the most common species seen in this role; only dogs (and miniature horses) are recognized as assistance (“service”^1^) dogs under the ADA, although other species may qualify as assistance animals in housing or air travel regulations.

Assistance Dogs International (ADI), a well-regarded organization that accredits non-profit facilities that train and place assistance dogs, recognizes three types of assistance dogs: (1) guide dogs (for the blind and visually impaired), (2) hearing dogs (for the deaf and hard of hearing), and (3) service dogs (for people with all disabilities other than those related to vision or hearing) (28). Between 1975 and 2015, Canine Companions for Independence (one of the largest US organizations placing assistance dogs in homes, primarily service dogs for mobility support) has placed ~5,000 dogs (27). The Seeing Eye, a guide dog organization, reports partnering over 17,000 people with guide dogs since the organization's founding in 1929, with ~1,770 of their dogs currently working in North America (29). Guide Dogs for the Blind, another major organization training and placing guide dogs, reports partnering more than 14,000 teams since their founding in 1942, with ~2,200 guide dogs currently working in North America (30). The number of roles that assistance dogs fill is also increasing, and the rate of dog placement is accelerating (27). For example, dogs are now being partnered with individuals on the autism spectrum or who are experiencing psychological issues such as anxiety or PTSD, or working in a human medical capacity (as cancer-detecting dogs, seizure-alert dogs, diabetic alert dogs, and similar).

Emotional support animals with their handlers who have disabilities are provided access to housing (under the U.S. Dept. of Housing and Urban Development) and transportation (U.S. Dept. of Transportation) for reasonable accommodation. These animals are not trained to perform tasks, but provide emotional support and companionship to their owners who have disabilities, including anxiety or other psychological issues (31). For more information on the different types of assistance animals, rights of access, and more, see the American Veterinary Medical Association's 2017 whitepaper on this topic (11). It is clear from these increases in the number of working assistance dogs and emotional support animals that veterinarians will likely see them increase in their practices as patients (4).

Another category of animals working in supportive roles are therapy animals managed by handlers without disabilities. These animals have no special legal access. Usually these are dogs, specifically trained, tested and registered to work in hospitals, nursing homes, schools or other institutional settings to comfort residents and facilitate interventions. These human-canine pairs may be registered with Pet Partners (https://petpartners.org/) or Therapy Dogs International (https://www.tdi-dog.org/), as sometimes required by institutions, but these registrations provide no special legal access. Pet Partners offers online courses for AAT handlers in reading canine body language and infection prevention and control which meet the recommendations from the Society for Healthcare Epidemiology of America for “Animals in Healthcare Facilities” (32).

### Guidelines and Implications for the Veterinarian:

**Figure 1:**
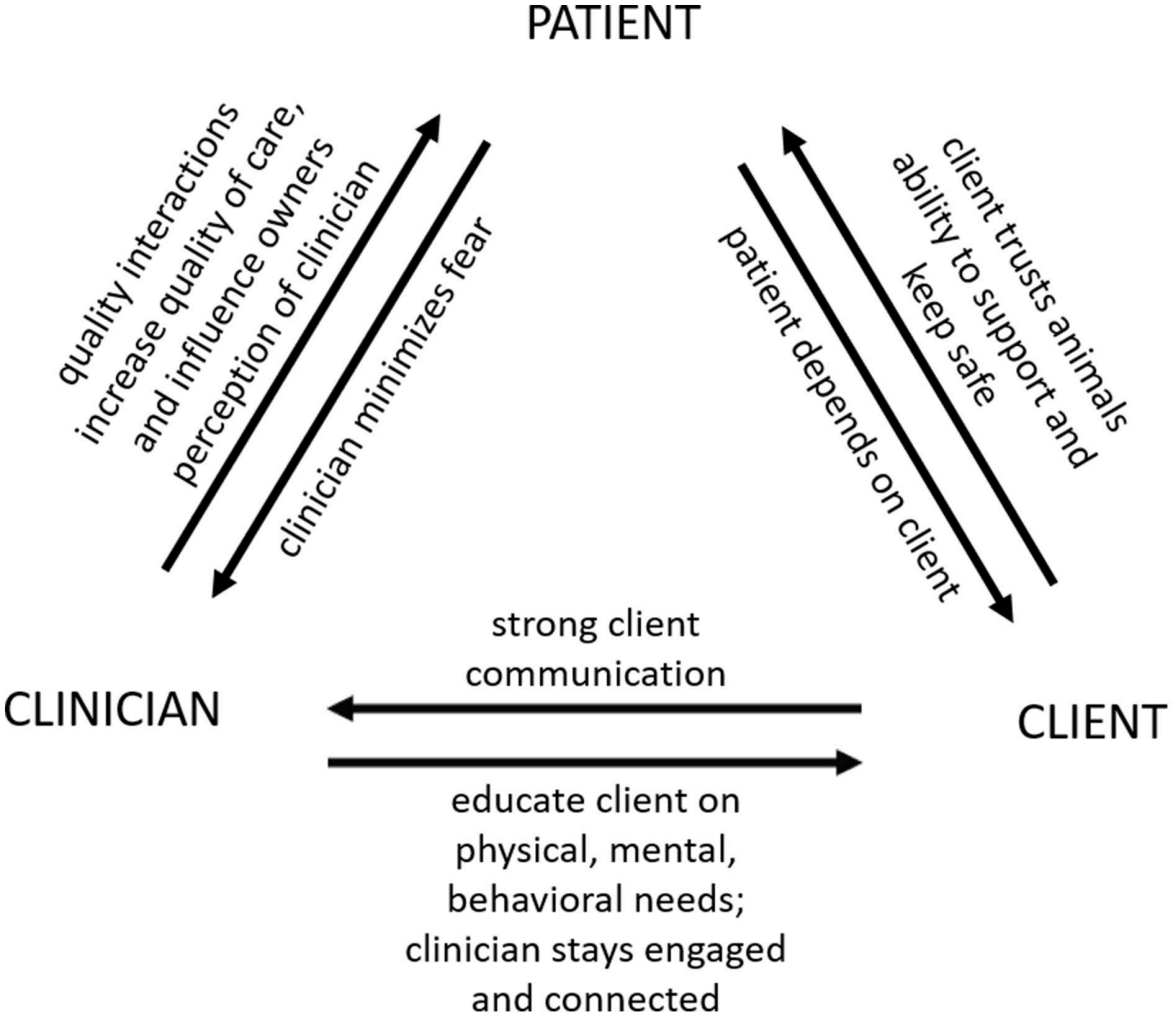
The clinician-client-patient triad in veterinary medicine (credit: A.P. Thigpen;^©^Emma Grigg & Lynette Hart 2018).

For veterinarians, and as with all clients, the primary goal in working with clients who have a disability should be to enhance comfort, engagement and adherence of the client, in order to ensure the best possible care for the patient (5, 33). In the practice of veterinary medicine, there is a triad of relationships at play: the bond between the client and the animal, between the clinician and the patient, and between the clinician and the client (Figure 1). All three relationships are important for optimal care. Bond 1, Client-Animal: Given the degree to which companion and assistance animals depend on their human caretakers, maintaining the bond between owner and animal is important for their wellness (34). In cases where the pet is an assistance animal, part of this bond may be the trust that the human partner feels with respect to the animal's ability to do the job: to support them and keep them safe (4). The veterinarian often needs to play the role of educator, as well as medical practitioner, to ensure that the human client is sufficiently knowledgeable about the animal's species-specific physical, mental and behavioral needs. Without this knowledge on the part of the caretaker, the risk of suboptimal welfare for the animal is high. Bond 2, Clinician-Patient: The quality of the clinician's interactions with the animal can influence quality of care, and the owner's perception of the clinician. Low-stress handling approaches (35, 36) are recommended for minimizing patient fear, putting the least amount of strain on this bond feasible in this setting. Bond 3, Clinician-Client: To provide the patient the best quality of care for life, it is important for the veterinarian to build and maintain this bond, to engage and stay connected to the client (37). A wide range of literature exists on strengthening client communication in veterinary practice [e.g., (38)]. All three bonds need attention and maintenance, for the working relationship to be a success.

To increase access to services, Sandler (39) recommended considering offering discounts on services, or special payment plans, to assistance dog partners; this recommendation could be extended to all clients with disabilities, given the income inequities noted above. As Eames and Eames (40) note, given the financial challenges faced by many people living with a disability (7), it is likely that cost of care, including veterinary care, is an obstacle for obtaining and living with an assistance dog for many of these individuals. This was confirmed in a small 2008 study of guide dog owners, when cost of maintaining the dog was noted as a significant concern (41). Pet insurance plans are becoming more commonly available, and are offered both by large veterinary practices and private insurance firms; such plans may benefit clients working with limited income, and/or with the costs of maintaining a working assistance or therapy dog. Websites comparing pet insurance and payment plans are available online, such as at ASPCA Pet Insurance (https://www.aspcapetinsurance.com/research-and-compare/compare-plans/compare-pet-insurance-plans/) or via Nationwide (https://www.petinsurance.com/comparison). The largest assistance dog training facilities sometimes offer annual stipends for veterinary care. Organizations such as the International Association of Assistance Dog Partners (IAADP) provide a wide variety of resources to human partners of guide, hearing and service dogs, and some preventative treatments (such as flea and tick prevention, glucosamine) to dues-paying members.

The remainder of this paper will focus specifically on building successful partnerships with clients who have disabilities, and/or when the patient is an assistance dog. Experts in this field suggest that allotting a small amount of extra time, effort and communication skill can greatly enhance the visit of a client with a disability, thereby improving the quality of care for the patient and the satisfaction of the client (4, 33). In the clinic, two specific target areas to focus on for building successful working relationships with clients with disabilities are: (1) orientation and assistance, and (2) successful communication, promoting client adherence (4).

## Actionable Recommendations

### Orientation and Assistance—Overview

**Figure 2:**
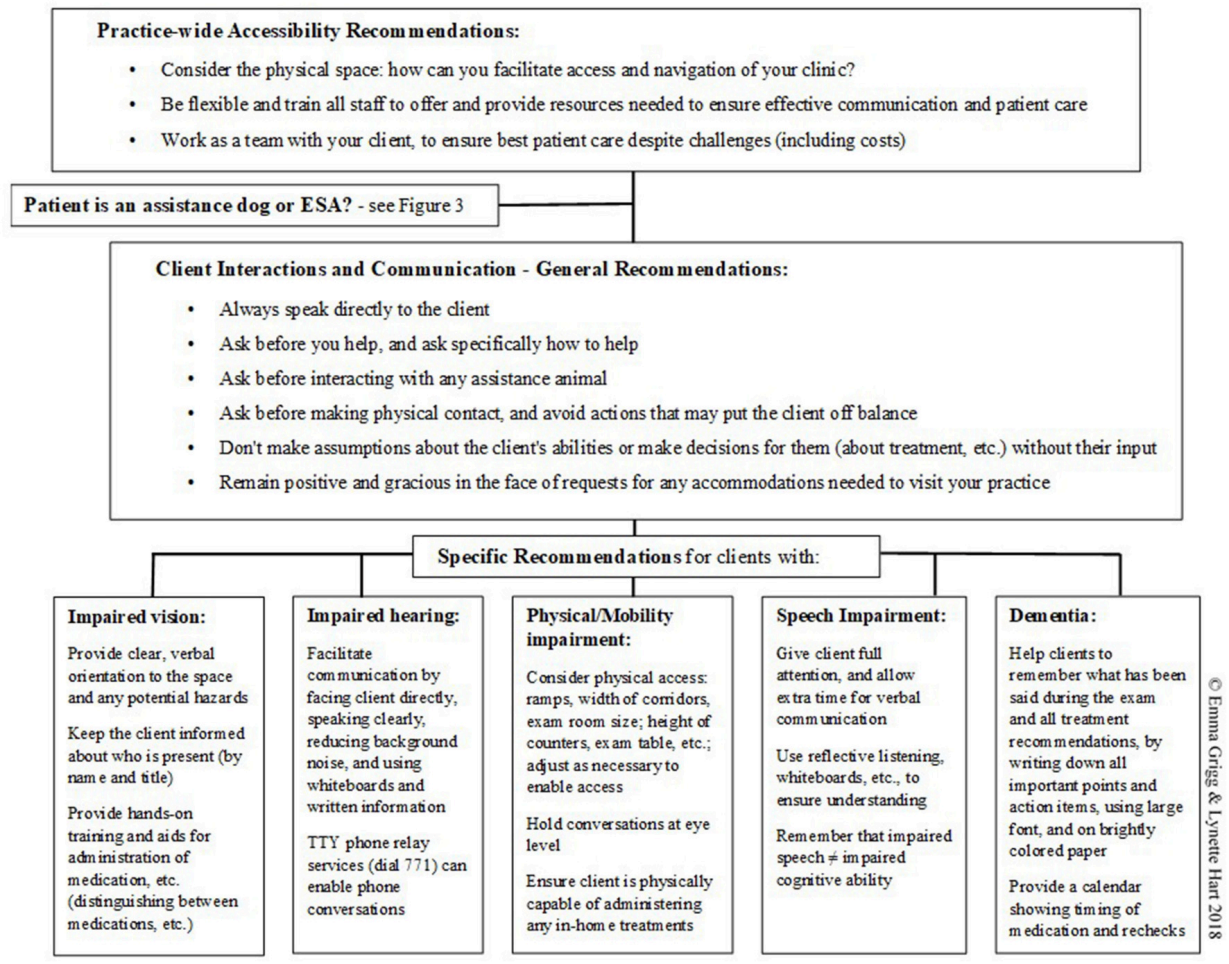
Summary of recommendations for working with clients with disabilities. Detailed recommendations and references are provided in the text (^©^Emma Grigg & Lynette Hart 2018).

Two important overarching goals to address in order to ensure an effective working relationship with clients with disabilities are: (1) the physical space (accessing and navigating the physical space safely), and (2) access to resources (ensuring the client has access to resources needed to make the visit a success) (4). Client needs will vary by disability; when in doubt about the best approach or accommodations needed, the clinician should ask the client. Veterinarians should be aware that two clients with the same or similar disability may use different adaptive techniques, and cater to the adaptive technique used by that client (33, 40, 42). Reasonable accommodations are required by law (43). A brief summary of these recommendations can be found in Figure 2.

Basic etiquette recommendations for interacting with clients with disabilities include the following (6, 10, 33):

1. Always speak directly to the client (not to the helper, translator or companion, if present), and maintain eye contact while speaking.
2. Ask before you help; offer help only if the client appears to need help. If the client does want help, ask how to assist before acting.
3. Similarly, if the client has an assistance dog with him, always ask before interacting with the dog in any way, particularly if the dog is actively working (e.g., in a harness, with the client holding the harness).
4. Be sensitive about making physical contact, and avoid actions (such as grabbing the client's arm) that might put the client off balance. This caution extends to personal equipment such as wheelchairs, canes, and scooters, which are often considered part of personal space.
5. Don't make assumptions about the client's abilities, or make decisions (about treatment options, for example) for them without their participation. Remember that a physical disability is not synonymous with a mental or cognitive disability, and the presence of one is not necessarily an indicator of the presence of the other. The client is the best judge of what she can or cannot accomplish.

A final etiquette recommendation very relevant to veterinary visits is to: (6) respond graciously to requests for accommodations needed to visit the practice. Such accommodations may require changes to the way the practice usually works, but staff should be flexible: a positive experience at the practice will make them more likely to return for future visits, and perhaps they will spread the word with others about the excellent service that they received (6). The improved quality of visit experienced by the client may also have a positive impact on quality of care the patient receives, for example by maintaining consistency of care through repeat visits (44).

Meeting these two overarching goals will require staff training. One of the most commonly-cited problems encountered by patients with a visual impairment when accessing human medical offices was the lack of staff training to understand their needs, according to a 2012 study of guide dog owners across 19 EU member states (4, 45). Train all staff with the goal of building a culture of sensitivity, understanding, and kindness; Paul (42) suggests asking oneself, “What would I be willing to do if this person was a family member or friend?” Many well-meaning staff may feel awkward in the presence of clients with a disability, if they are unsure of how to behave and worried about inadvertently offending the client. At minimum, ensure that all staff are aware of the basic etiquette recommendations listed above. Continually work on improving listening skills, and remember that every client may have special needs from time to time (46). Confront any prejudices or misinformation amongst staff members about people with disabilities, as prejudging clients can interfere with successful communication and care (46). Remember that the mission as practitioners is to make life better for pets and their people (42). Put standards of practice in place to ensure that clients with a disability receive the same quality of care as all other clients. At staff meetings, discuss specific strategies designed to ensure access and availability of resources necessary to make these client visits a success (10). When appropriate, instruct staff to proactively ask what accommodations or assistance the client will need during the visit, to ensure that the client's and patient's needs are met (4). Empathy alone is not enough; for patient care to be effective, the working relationship with the client must also be effective (9). If some staff members are particularly patient or experienced with individuals with special needs, request that they be present in the exam room during these visits (42). Using checklists (listing steps to take before and during a visit, and resources to have on hand) can be very helpful; a sample checklist is available online (46); checklists can be modified or expanded as appropriate for the specific clinic and clientele. The summary provided in Figure 2 may also be a useful reminder of key points for staff.

Schedule appointments at slower times of the day, to allow ample time for working with clients with disabilities (42). Consider allowing flexibility in exam times, scheduling the client to arrive within a window or block of time, rather than a set appointment time. If unable to drive themselves, many of these clients may rely on taxis, ridesharing services or public transportation, making meeting exact appointments with their pets challenging (33, 39). Note, however, that it is not appropriate to charge more if these appointments take longer because the client was disabled; any action that disadvantages a person based on his or her disability would be considered discriminatory (43).

In order to ensure that clients with disabilities are able to safely and comfortably access the physical space, assign at least one parking space for these clients, with highly visible signage. Have at least one entrance accessible to wheelchairs, ensure ramps, and hallways are free of obstructions, and post easily-understandable, highly visible signage with good contrast (4). If feasible, help to arrange transportation as needed for clients who cannot drive, including arranging for delivery of medications, supplies, etc., if needed (46). Be flexible with use of the existing clinic space. For example, if the exam room is too small to accommodate a client's wheelchair, find another suitable space where the exam can be completed; this is vastly preferable in most cases to separating the pet and the client (or worse, separating the client from his or her assistance dog). Consider having seats with arms in the reception area, as these make it easier for senior citizens and those with mobility issues to sit down and get back up (6, 46). Good lighting; marked visual contrast between floors and walls, and on staircases; handrails mounted in appropriate locations; and use of large font on all written materials will be appreciated by many clients with disabilities (4, 46).

### Successful Communication:

Two primary areas to focus on to ensure successful communication with clients with disabilities are (1) finding modes of communication that work best for the individual client, as disabilities vary and thus, most effective means of communication will vary; and (2) given challenges that exist in veterinary medicine for achieving optimal communication with, and adherence by, clients without a disability, it is important to acknowledge that additional challenges can exist when working with disabled clients (4, 33, 37).

It is important to acknowledge all clients as individuals. Eames and Eames [(33); p. 1] stress a “People First” concept, noting that “these clients (with disabilities) are people first. They are not their disabilities.” This is perhaps most evident in the language we use to refer to, communicate with, clients with disabilities. Put the person first: referring to a “person with a disability” is preferable to “disabled person” (6). It is also generally acceptable to refer to specific disabilities, e.g., person with hearing loss, or person with Alzheimer's disease. Avoid outdated terms, like “handicapped” or “crippled”; negative terms like “suffering from”; and euphemistic terms like “physically challenged” or “differently abled,” which many individuals with disabilities find patronizing (6). It is generally best to respect the client's privacy and not ask directly about their disability; however, Cohen (6) notes that many people with disabilities are not bothered by children's natural curiosity and don't mind answering their questions.

Recommended theoretical approaches to client communication in the veterinary field have evolved from compliance (a more traditional approach in which the clinician simply tells the client what treatment to follow, and which is now considered too paternalistic toward the client), to concordance (in which the clinician and client should come to an agreement about the treatment approach), to adherence (in which the client takes an active—vs. passive—role in the treatment and care of their animal) (47). Adherence is defined by the World Health Organization (WHO) as “the extent to which a person's behavior (taking medication, following diet, and/or executing lifestyle changes) coincides with agreed recommendations from a health care provider” (48). This concept describes the relationship between the patient and health-care provider as a partnership drawing on the abilities of each; the WHO notes that “the literature has identified the quality of the treatment relationship as being an important determinant of adherence” [(48); p. 3]. Adherence relies on designing a treatment plan around the client's lifestyle, rather than the other way around, and the term itself implies the tenacity that clients will need to follow the treatment regimen (47). In addition, more “relationship-centered” or “family-centered” (49) approaches may be more suitable for meeting the needs of companion animal patients, given the role of the human household members in the health and well-being of companion animals. For these reasons, and in the absence of research into outcomes for different communication approaches when working specifically with clients with disabilities, adherence may be an optimal model for working with these clients.

To facilitate adherence to the recommendations for care and treatment, elicit the client's perspective, as clients are more likely to follow treatment recommendations that they understand and endorse. Communication is a two-way street; identify barriers to successful treatment of the patient, and work with the client to develop a plan that will work for them (37). Barriers could include logistical challenges, physical or mental challenges, incomplete understanding about exactly how to administer the treatment, lack of conviction that the treatment is necessary, and/or discomfort with the procedures (clients with a fear of needles, for example, will have trouble using needles for administering treatments at home). This is particularly important if/when the client is already facing significant daily challenges associated with a disability. Do not assume that the client has understood; work with the client to ensure mutual understanding. After discussing treatment recommendations with the client, Abood (37) recommends assessing client conviction and confidence, two important factors influencing the client's level of preparedness and commitment to adhere to the clinician's recommendations:

*Assessing conviction*: Ask the client, “On a scale of 1–10 (with 1 representing completely unnecessary, and 10 representing completely essential), how valuable do you think (this treatment) is to helping (patient's condition)?” A low rating by the client indicates that they are not convinced, and/or not ready to take action.
*Assessing confidence*: Ask the client, “On a scale of 1–10, how confident are you that you can carry out this treatment plan?” Here, a low score indicates that adherence to the recommended treatment will be low.

As noted in the Basic Etiquette section in *Overview*, above, staff should always address the client directly, even in cases where a translator is present. If the client is seated or using a wheelchair, the clinician or staff member should sit down so that they are at eye level with the client for any extended conversations such as history-taking or discussion of treatment options.

### Specific Recommendations (by Disability)

#### Working With Clients who Are Blind or Have Impaired Vision

Upon entering a room (such as the reception area or waiting room), provide a clear verbal description of the size of the room, location of available seating, and other animals present; even if the client has been to the practice before, seating and presence of other animals will vary, and clients with visual impairment will not be able to read warning signs of aggression in other animals present (4). Offer physical assistance, but if declined, still provide a verbal orientation to the room. If the client has not been to the practice before, consider offering him a tour of the facility (6). Remember to always speak directly to the client.

When it is time for the appointment, staff should approach the client rather than calling their name from across the room; introduce themselves by name and title, and ask whether the client would prefer guiding or to follow their guide dog, if present (4, 33). If the client has a guide dog, it is best to walk on the opposite side as the dog. When guiding, the person guiding should stand on the client's right and offer his or her left arm (50); allow the client to take the arm of the person guiding, rather than grabbing the client's arm, pushing or pulling, as this may put the client off-balance. While walking, describe the path of travel, including any obstacles such as stairs (up or down), furniture or fixtures, doors (noting whether the door is open or closed, and if closed, which way the door opens, in or out); use approximate number of steps to indicate distance (4). The person walking with the client should warn her of an obstacle in her path, be specific and use non-visual warnings: “Look out” is less helpful than “Be careful of the step up two paces in front of you” (6). Ensure that clear glass doors and panels are clearly marked at the appropriate height (4).

If meeting the client in the exam room, the veterinarian should introduce herself and other staff present by name and title; similarly, indicate if/when anyone leaves the room, so that the client does not end up unknowingly talking to someone who is no longer present. Indicate locations of any possible hazards, like the exam table, to prevent injury to the client (when bending down to reach their pet, for example) (33). The clinician should narrate each step of the exam verbally, including weight, temperature, and the like; avoid using descriptors relying on vision, such as “over here” and “this,” as these generally do not provide useful information to a client with vision impairment. Audible cues should be used when appropriate to indicate location (such as tapping the chair or exam table where staff would like the client to stand) (33).

When dispensing medication or other treatments, clients with limited vision may need a hands-on demonstration in the exam room of how to administer medication, change dressings, etc.; allow the client time to practice this to ensure comfort with the procedure. If the client has brought a helper along who will be assisting with treatments, the helper should receive this training as well. When possible, staff should ask if the client would prefer liquid or pills, and ask if they prefer easy-open caps (46). It is best to use pre-measured doses whenever possible (for example, split pills ahead of time), use notched syringes to indicate proper levels of liquid to administer, and if possible, consider dispensing extra medication to allow for spillage (9). Offer to read aloud any written information on the medication or product packaging. If multiple medications are dispensed, use different sized and/or shaped containers to allow the client to differentiate between medications; alternately, rubber bands around one of the containers can help (33, 46).

#### Working With Clients who Are Deaf or Hard of Hearing

When speaking to the client, staff should always face him directly, speak clearly and expressively (gestures and facial expressions can be helpful in providing context for what is being said), speak directly to the client (not to an interpreter, if present), but should not raise the volume of their voice above normal levels unless requested to do so by the client (33, 51). Do not cover any part of the face (e.g., with hands, a pen, clipboard, or tablet) while speaking (6), as this can impede the client's ability to read lips. In the veterinary clinic, clients may be stressed or distracted; be sure to get the full attention of the client before speaking (52); tap the client gently on the arm or shoulder if necessary (6). Reduce background noises, ask straightforward questions, and allow extra time for the client to understand and respond (46). Note that the staff may need to repeat themselves, but should be patient in pursuit of effective communication. It is still important to explain verbally what is being done throughout the exam, so that the client understands what is being done to their animal (51). Use of a whiteboard in the exam room can be very helpful by allowing back-and-forth written communication and providing visual aids to help explain medical terms, procedures, or treatments (42).

When a phone is used for scheduling appointments or follow-up, veterinary staff should be aware that telecommunications relay services are available to assist in phone communications with clients with impaired hearing (33). These relay services can be used by clients relying on a teletypewriter (TTY) to make and receive calls, and allow these clients to call businesses that do not have a TTY available. If staff receive a call through the telecommunications relay, the operator will identify it as such (6). In the US, this service is accessed by dialing 711 to connect to a trained operator; more info on this service (and similar services available using internet or video) can be found at: https://www.fcc.gov/consumers/guides/telecommunications-relay-service-trs. In the age of smart phones and electronic tablets, it may be easier for many clients with hearing impairments to communicate by text messaging, web-based communication or email.

#### Working With Clients With Physical/Mobility Impairments

The capabilities of clients with physical disabilities can vary widely, and may include mobility difficulties, impaired motor skills or hand strength, or other issues. Ensure that the recommendations designed to facilitate access to the physical space, described in the *Overview* section above, have been reviewed and addressed as necessary. It is essential to personalize the treatment plan so that the client is physically able to adhere to it (including being able to open medication bottles, prescription diet cans, and the like); if a helper is present, enlist their help and ensure that both client and helper are comfortable with the techniques required (33). See also “Assessing Conviction and Confidence,” above (37). Some clients may benefit greatly from in-home (vs. clinic) visits for veterinary care, if this service is or can be made available, even if just for help with ongoing treatments (giving pills, administering topical ointments, changing dressings, etc.) (46, 51).

For clients using wheelchairs, consider the height of signage, counters (in the reception area, for example), and the exam table, particularly when the client is expected to be able to see what is happening (9). Don't make clients “talk to the wall” of a high reception desk when checking in; instead, staff should come around the counter to speak directly to the client, and take a seat for longer conversations. If it is difficult for the client to see his pet during the exam, or when the patient is an assistance dog, the veterinarian should consider doing the exam on the floor rather than on the exam table. For some clients with mobility issues (those who rely on service dogs, for example), shaking hands might be difficult, so follow the client's lead during greetings (51). Ask the client what assistance or additional accommodations they will need to make the visit a success (33). Note that some wheelchair users appreciate being pushed over difficult surfaces (such as carpeting), but others do not; when help appears to be needed, always ask before pushing an occupied wheelchair (10).

#### Working With Clients With Speaking Difficulties

Veterinarians and staff should give the client their full attention during conversations. As noted above, it is important to remember that impaired speech is not synonymous with impaired cognitive ability (33). Be patient and allow the client extra time to communicate questions or concerns; resist the temptation to interrupt or attempt to finish the client's sentences, even if trying to be helpful (6). Many of the recommendations for facilitating successful communication with a client with hearing difficulties can also be beneficial for clients with speaking difficulties, such as reducing background noise and using a whiteboard to allow written communication. Staff should not pretend to understand the client if they have not; instead, they should ask the client to repeat himself or to write down the information on the whiteboard. Practice reflective listening: summarize what you have heard, and look for confirmation or clarification from your client (33). For example, when possible, ask short yes or no questions that can be answered with a nod (for example, “Do you feed Max once per day?” is easier to answer than “Tell me how often you feed Max.”) In most cases the client will appreciate the staff's effort and interest in understanding what he has to say (6, 33).

#### Working With Senior Clients With Memory Loss or Dementia

A primary challenge for these clients is remembering what is said during the exam, including treatment recommendations. Staff should write down all instructions, preferably in large/bold font and on brightly colored paper, and ask the client to post the instructions prominently in their home (such as on the outside of the kitchen refrigerator). Make a calendar for the client to take home with dates when medicines and/or upcoming recheck visits are due (46). When necessary, use reminders and follow-up by phone, and if possible, help make arrangements with a family member or carer to ensure that the treatment recommendations are followed. Clients with dementia may also suffer from other physical/mobility or sensory impairments, so the recommendations above on these topics may need to be implemented when appropriate. In addition, clients with dementia may become anxious in the veterinary clinic, and require patience and calm interactions. A number of recent studies and white papers [reviewed in Kruger and McCune (53)] have documented mental health and quality of life benefits for seniors living and interacting with pets (54, 55), and it thus seems advisable to support pets living with their senior owners for as long as quality of care can be maintained. Veterinarians can play a role here by more actively monitoring the health of patients living with these clients, for example by using more frequent follow-up calls or scheduled visits; it may be particularly useful to identify a family member or caregiver for the client who is able to assist with care for the pet and/or communicating with the veterinary practice.

## Assistance Dogs as Patients in the Veterinary Practice

**Figure 3:**
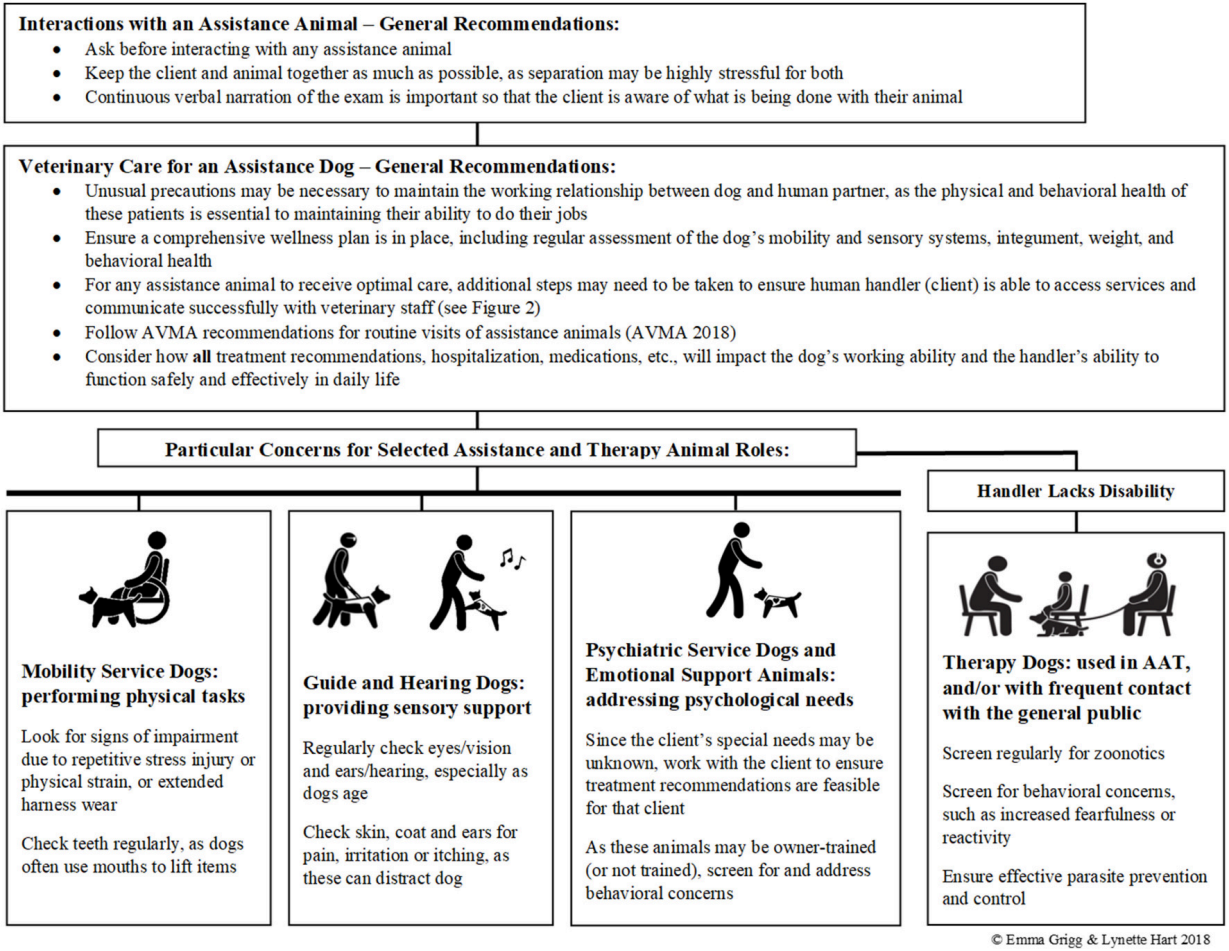
Brief recommendations for assistance and therapy animals as veterinary patients. Detailed recommendations and references are provided in the text (^©^Emma Grigg & Lynette Hart 2018).

Assistance dogs can provide significant physical, psychological, and social benefits for persons with special needs (19, 23, 24, 26, 27). Veterinarians play a crucial role in maintaining this working relationship, primarily by maintaining the wellness of the animals involved (56). The physical and behavioral health of these dogs is essential to maintaining their ability to do their jobs, and in this sense, the veterinarian is also indispensable to the human partners of these animals, who depend on their dog's abilities to function in their worlds (9). In addition to their value as companions and helpmates, these dogs are very valuable animals in the monetary sense; the average cost to raise and train an assistance dog can range from $15,000 to $50,000 (57, 58). Unusual precautions may be necessary to maintain the working relationship these dogs have with their human partners (39, 58). In some cases, for example when the dog has been owner-trained to assist with their own disability, veterinarians may provide the only professional oversight for the welfare of these animals. For any procedure, medication, hospitalization, etc., veterinarians need to consider how this will impact the dog's ability to do his/her job, and thus how it will impact the dog's human partner. This requires an understanding of exactly how the dog helps the human; what specific tasks does the dog do? If not sure, the clinician should ask the client to ensure that understanding of the dog's needs (9, 40); questioning what tasks the dog has been trained to perform is permitted under the ADA, as this question is not considered to violate the rights of people with disabilities (59). A brief summary of recommendations for treating assistance and therapy animals as patients can be found in Figure 3.

A comprehensive wellness plan should be in place; these dogs rely on excellent senses and mobility to do their jobs, and thus regular examination and assessment are important (56, 58). The wellness program should consider the life stages of the dog (60), be flexible and tailored to the needs of the individual dogs and clients. Up-to-date records should always be maintained, and wellness visits should be regular enough to detect any signs of physical and/or behavioral decline (56). Migday (58) recommends that visits should occur as frequently as every 2 months, if possible, particularly as these dogs age. Mechanisms should be in place (scheduled appointments, for example) to ensure that the veterinarian is able to regularly complete these wellness checks. AVMA (56) guidelines for the care of animals involved in animal-assisted interventions of any kind stress that wellness includes both the physical and behavioral health of the animal.

Recommendations for routine visits include (56):

- Vaccination
- Parasite control and prevention
- Screening for common diseases and conditions as applicable to the dog, region, etc.
- Behavioral evaluation
- Preventative medical, dental, and nutritional care
- Preventative behavioral care (education about normal behaviors, reading body language, and the importance of enrichment)
- Assessment of genetic health as appropriate

Given the physical nature of the work that many of these dogs do on a daily basis, regular, and careful assessment of eyes/vision, ears/hearing, hips, teeth (as many of these dogs regularly pick up items for their human partners using their mouths) and feet is important (40). Clinicians should be vigilant for signs of impairment due to repetitive stress injury and/or excessive physical strain, such as pulling wheelchairs or opening doors, and be familiar with the physical strain put on the dog by typical harnesses, such as may be caused by asymmetrical torque placed on guide dogs by handlers holding on to the harness handle (61–64). Skin, coat, and ears should be checked regularly and treated as needed for pain, irritation or itching, as these can be very distracting to the dog and may interfere with focus and working ability. Skin should be checked for lesions caused by rubbing or pressure sores from harnesses or backpacks (58). Coat maintenance may be difficult for clients with disabilities, so the dog's coat should be checked regularly for mats, particularly in areas like the groin, or behind the ears (58). Healthy weight should be maintained through diet and regular exercise, to sustain peak physical and mental condition. Wakshlag and Shmalberg (65) recommend keeping most assistance dogs between a body condition score (BCS) of four and five (using a nine-point scale) to prevent fatigue and joint-related problems associated with carrying excessive body weight. Assistance dogs' diet and activity level will be largely determined by the lifestyle of their human companion, and many of these dogs may have nutritional needs similar to active companion dogs, but they should be regularly monitored for weight gain that may interfere with their working ability (65). Assistance animals and animals used in animal-assisted interventions should be screened regularly to reduce bi-directional risk of zoonotics transmission (56, 66). For example, in a cross-sectional study of 102 dogs visiting hospitals in Ontario zoonotic agents were isolated from 80% of the dogs, primarily *Clostridium difficile*; others identified included *Escherichia coli, Salmonella*, and *Giardia* antigen (67). Chomel (68) provides an overview of zoonoses in dogs and cats, including a discussion of recent studies of zoonoses reported in dogs fed a raw meat diet.

During the exam, best practice is to keep the dog and client together as much as possible, as either or both may become highly anxious when separated from their partner (40). Verbally narrating the exam can reduce client anxiety (particularly for clients with impaired vision) and improves communication; when the client is using a wheelchair, examining the dog on the floor will allow the client to see what is being done (40, 51). Targeted recommendations are listed above for working with clients with specific disabilities.

### Behavioral Welfare of Assistance Dogs, Therapy Dogs and Facility Dogs

As noted in the AVMA (56) guidelines, wellness encompasses both the physical and behavioral health of the animal. Working as an assistance animal can be a demanding job, and there may be many potential sources of chronic stress for these dogs [reviewed in Serpell et al. (63)]. Public concern for the wellbeing of these dogs while working is increasing. Bremhorst et al. (69) described risk areas for welfare of assistance dogs, including lack of sufficient time off, ergonomics of harnesses, and weight gain, and noted that veterinarians need to look for signs that problems like these exist. Education of clients about the behavioral needs of these dogs is crucial: clients should be familiar with the behavioral signs of stress in dogs and use rewards-based training only. Ziv (70) reviewed impacts of training approach on welfare of companion and working dogs (primarily dogs trained for military and law-enforcement work), and concluded that use of aversive training methods such as positive punishment and negative reinforcement can jeopardize both the physical and mental health of dogs, and that there was no evidence that aversive training methods are more effective than positive reinforcement-based training. LaFollette et al. (71) found that use of positive reinforcement or “bond-based” training methods for PTSD service dogs was associated with more positive outcomes for the veterans (such as higher perceived closeness to the dog and more attachment behavior), while use of positive punishment was associated with more negative outcomes (such as more fear and less trainability). Serpell et al. (63) also recommend use of only rewards-based training methods for training assistance dogs. In addition, clients working with assistance dogs should understand the importance of downtime and play, and of monitoring the dog during interaction with others to avoid inadvertent harm by children, or others with disabilities (69).

A number of recent studies have investigated stress levels in working assistance dogs. These studies have focused primarily on dogs working in animal-assisted-interventions, and overall the results are encouraging, although sample sizes are often small. Palestrini et al. (72) looked at heart rate and behavior of one experienced service dog over >20 20-min sessions working as a therapy dog, and reported no physiological or behavioral signs of stress, fatigue or exhaustion. Glenk et al. (73) and McCullough et al. (74) observed dogs used in therapy sessions in very different settings, and both reported that dogs in their studies were not stressed by repeated work in these sessions, based on behavioral and physiological (cortisol) indicators. King et al. (75) monitored stress in dogs working in animal assisted therapy in varied environments using cortisol measurement, and assessed effect of a quiet play time-out session during work shifts. That study reported no significant impact of the time-out session, but did note a trend of increased cortisol from baseline to 1-h into the work shift (75). They also reported more behavioral signs of stress in young (<6 years old) dogs and inexperienced dogs, vs. older and/or more experienced dogs. Haubenhofer and Kirchengast (76) looked at cortisol in pet dogs working in animal-assisted therapy, and reported that cortisol levels were significantly higher on working days vs. control days; however, as this study did not measure behavioral indicators, it is unclear whether this was negative stress or positive excitement, as the authors themselves note. Additional research would be beneficial to understand if, when, and in what ways assistance dogs experience work-related stress.

Although stressors on assistance dogs will vary according to the type of work that they do, a widely-accepted baseline for assessing and improving animal welfare is the Five Freedoms concept (77). This concept has been recommended as a useful tool for working toward wellbeing and good quality of life for working, assistance, and companion dogs (62, 78, 79), and is endorsed by organizations such as the American Society for the Prevention of Cruelty to Animals (ASPCA) and the Association for Shelter Veterinarians (ASV). The Five Freedoms, originating from a 1965 report (80) on production animal welfare in the UK, are as follows:

1. Freedom from Hunger and Thirst (by ensuring ready access to fresh water and diet to maintain health and vigor).
2. Freedom from Discomfort (by providing an appropriate environment including shelter and a comfortable resting area).
3. Freedom from Pain, Injury or Disease (by prevention or rapid diagnosis and treatment).
4. Freedom to Express Normal Behavior (by providing sufficient space, proper facilities and company of the animal's own kind).
5. Freedom from Fear and Distress (by ensuring conditions and treatment which avoid mental suffering).

Working with the client, veterinarians can conduct a brief “Five Freedoms Test” (81), comparing the dog's current health and lifestyle with these basic minimums, and paying particular attention to the last two freedoms (as these are more challenging for many humans to understand and provide for their dogs). Education of the client about behavioral needs of domestic dogs (e.g., having an outlet for natural behaviors, spending time with conspecifics, sufficient physical, and mental exercise) and about canine body language are important components of caring for these dogs. Veterinarians should inquire about behavior as an indicator of welfare during each exam, bearing in mind that some clients' disabilities may interfere with their ability to read canine body language, and should address any deficiencies in the dog's care that might be contributing to decreased welfare (65). In particular, fear and anxiety can compromise not only the assistance dog's welfare, but also the dog's working performance (82, 83), and can lead to early withdrawals from working roles (84) [reviewed in Rooney et al. (85)].

### Medications, Sedation, Anesthesia, Hospitalization and Emergencies

In order to maintain the working abilities of assistance dogs, the clinician should always consider how any of these procedures and/or treatment options will impact the dog's physical, mental and sensory competence, and for how long. Medications that cause sedation (e.g., antihistamines), mental “dullness,” vomiting or diarrhea can impact the dog's ability to work effectively [reviewed in Sandler (39)]. Even a commonly-prescribed medication such as a corticosteroid to treat itching or allergies can cause real problems for the dog's human partner, as a common side effect of these medications is a marked increase in the dog's need to urinate (39, 40, 86). The dog should be back to full working capacity by the time he leaves the clinic with his handler, as the client depends on the dog's abilities (40). It is important, in any case, to provide complete information about the dog's current physical and mental condition to the client. Eames and Eames (40) relate cautionary tales of when this information was not provided to the human handlers, for example in the case of a guide dog released into the care of his handler while still disoriented after suffering a stroke. Hospitalization should be reserved for the most serious cases, or when empiric treatment is not possible (39). Loss of the dog, even temporarily, can represent a significant logistical hardship for the client; if hospitalization is necessary, it may be better to discuss this in the privacy of the exam room, as this may be a sensitive issue for clients who depend on their dog (4). The veterinarian's responsibility in these issues is to both the animal and the client, however, and this can present contradictory goals (87). If the animal's well-being (distinct from their continued ability to work as an assistance dog) requires hospitalization, the veterinarian should work with the client to find a way to get the dog the necessary treatment with the least possible hardship for the client (88). If the client has to leave the clinic without his dog (for example, in the case of an unexpected emergency), ensure that he has safe transportation home. Guide dogs should not return to work for 24 h after a sedative or anesthetic has been administered; in these cases, the staff should notify the client in advance so that he or she can arrange for alternate transportation back home (4). The extreme stress that the client may experience in an emergency situation may exacerbate existing communication challenges. Staff should be aware that getting necessary information may take longer than is desirable in such situations (52), and they should be prepared to be particularly patient with these clients during emergency visits.

### Aging, End of Life and Loss of an Assistance Dog

As these dogs age, veterinarians caring for assistance dogs should continue to track how the welfare and working ability of these dogs may be changing (57, 62). Many domestic dogs experience cognitive decline and physical impairments as they age, which can include symptoms such as disorientation, altered interactions with humans and other animals, sleep-wake cycle disturbances, house-soiling, and changes in activity levels (89); clinical signs that are often classified with the acronym DISHA Loss of previously-learned cues may also be evident (89), particularly in highly-trained assistance dogs. These changes can significantly impact working ability, and if detected by the owner or veterinarian, the dog's duties may need to be curtailed, or retirement for the dog considered (63). Recommendations for maintaining cognitive function in domestic dogs as they age include ensuring sufficient mental and physical enrichment, feeding a diet specifically formulated for senior dogs, and supplementation with antioxidants (90, 91) nutraceuticals (92), and/or medication such as Anipryl (L-deprenel; Zoetis) (93). Guidelines such as the American Animal Hospital Association's Senior Care Guidelines for Dogs and Cats (94) can assist in ensuring optimal physical and mental wellness in aging assistance dogs. Many guide dogs are retired due to problematic changes associated with aging (95), but the choice about when to retire an assistance dog will vary depending on the dog, the owner, and the type of work that the dog does on a daily basis. Veterinarians can assist with this decision-making process by providing ongoing information about the mental and physical health of the dog, and options for prolonging working ability and quality of life. Research-based recommendations for assisting these dogs to transition from working into retirement are lacking (69).

These dogs fulfill important attachment and caregiving needs for their human partners, and the loss or imminent loss of an assistance dog can be a source of intense grief for these clients (96, 97). Veterinarians and clinic staff should familiarize themselves with the ways in which client grief may be expressed, and may benefit from continuing education or additional training in helping clients cope with their grief, provided by a qualified counseling professional (especially if they feel uncomfortable working with clients in this situation, and/or if sufficient training was not provided by their veterinary school curriculum) (98). Clients who are already experiencing adverse events in other aspects of their lives will be particularly at risk of severe grief associated with loss of an assistance dog (99). Levels of stress at retirement of the dog are generally lower for clients who continue to live with the dog after retirement, or who are able to place the dog in a home of their choosing (99). In addition to the emotional impact, however, there are significant logistical challenges and added stress associated with this event (100). Loss of the dog represents a significant loss of independence and mobility for many clients, and in most cases, these impacts continue while the client applies for, waits for, and trains with a new dog (40). Veterinarians working with clients relying on assistance dogs should provide these clients as much information as possible on the timing of the illness, quality of life, and the ability to keep working, and assist with planning for transitions in any way relevant to their role in the veterinarian-client-patient triad. Some clients may benefit from working directly with a human medical professional trained in grief counseling, or by seeking support and advice from an assistance dog organization (100). If euthanasia is necessary, clients with disabilities may benefit from in-home euthanasia of the animal, which is not only less stressful for many clients and patients, but also eliminates the logistical challenges for the client in accessing the veterinary clinic.

### Special Considerations for Wellness of Kenneled Dogs

Numerous studies conducted to assess welfare of domestic dogs living in kennel facilities, and using behavioral, physical, physiological, and cognitive measures, have reported that these dogs may experience suboptimal living conditions, particularly when kenneled for longer periods of time (101–108). For example, in a study of 148 dogs at eight rescue shelters in the United Kingdom, kenneled dogs displayed behaviors commonly associated with frustration and depression for 8 weeks following admittance to a shelter (104). Two groups of beagles housed for 6 weeks in social and spatial restriction showed both behavioral and physiological signs of chronic stress (102, 109). Although these studies focus on companion, research/laboratory, or working (military) dogs, given the frequently reported associations between kennels housing and stress in domestic dogs, risks to welfare of assistance dogs should be considered when these dogs are living in a situation where they are regularly kenneled (such as during their training, or when working as therapy dogs in a residential facility) (63, 69, 78). Welfare of these dogs may be compromised due to numerous factors, including lack of exercise and/or control over their environment, confinement to a small area, high and/or unpredictable noise levels, and minimal social contact (78, 110, 111). Behavioral indicators of stress in these conditions may include salivating, panting, restlessness, lowered body posture, trembling, hypervigilance, and an intensified startle response, among others (101); these behaviors likely indicate the presence of fear, frustration, and/or internal emotional conflict (105). Changes in behavior, such as the development of repetitive and stereotypical behaviors (e.g., spinning, circling, jumping in place, excessive barking) are also associated with chronic stress due to kenneling, particularly when dogs are housed alone (103, 111). Note that not all dogs will show these negative effects, but the development of behavioral issues associated with fear and aggression may make dogs experiencing these issues unsuitable for use as assistance dogs. High levels of physiologic stress experienced in kennels can result in poor training performance, which may in turn negatively impact working performance (78). Veterinarians responsible for the medical care of dogs housed in such facilities should review the facility's management protocols for these dogs in light of the American Association for Shelter Veterinarians' Standards of Care document (79), which provides recommendations for all aspects of the care of kenneled dogs. Any signs of behavioral decline in these dogs should be investigated and addressed promptly.

The AVMA (56) guidelines for animal-assisted interventions recommend ensuring that, in addition to being physically healthy, animals serving these roles are behaviorally appropriate for the program (given the type of interactions between the dogs and people), and that animals are protected from being harmed by participation in the program. There should be a clear “chain of command” and identification of those individuals responsible and accountable for the care of kenneled dogs. Veterinarians should identify the specific person responsible for the animals' welfare (the “responsible person,” or RP) and all necessary contact information, and should communicate regularly with the RP in both the development and implementation of an optimal wellness plan for these dogs (56, 61).

### Invisible Disabilities: Working With Psychiatric Service Dogs and Emotional Support Animals

As noted above, the number of assistance animals is increasing, along with the types of roles these animals fill. This likely reflects the parallel changes in the way that humans view their companion animals (87, 112); increasingly, clients tend to view their animals as part of the family (113), and in one recent survey, 93% of respondents reported that they would risk their lives to save their pets (114). Companion animals are now regarded as beneficial to human mental health (12), and often now serve as assistance animals for those with “invisible” or “hidden” disabilities, as psychiatric service dogs or emotional support animals^2^. Invisible disability is a broad term that covers a wide range of physical and mental disorders; to be considered a disability under ADA, the disorder must substantially limit one or more of a person's major life activities, such as walking, seeing, sitting, breathing (43). Many invisible disabilities would meet this criterion, such as chronic pain, chronic fatigue, attention deficit hyperactivity disorder (ADHD), Asperger's syndrome, anxiety disorders, and clinical depression (to name just a few) (115).

This can put the veterinarian in a challenging situation, as they may only learn of the animal's status as an assistance or emotional support animal after the exam is completed, or treatment recommendations have been made (88). In fact, the veterinarian may never know about the disability if the client does not wish to share this information, but if present, any disability that interferes with normal life function may also interfere with the client's adherence and thus the success of treatment and care. In either situation, the best course of action may be to observe best communication practices such as those described earlier in this paper. The veterinarian should work with the client to arrive at a treatment plan to which the client can realistically adhere, given challenges in the client's life which may or may not be known to the veterinarian or her staff. It is important to accept the client's own descriptions of their ability or disability; as the term implies, many of these individuals may not “look like they have a disability.” Clinicians should familiarize themselves with state and national laws to establish what they can and cannot legally ask their clients about their disabilities and the status of their assistance animal (88, 116). While it is not generally appropriate (or legally permissible) to ask questions about the client's personal medical history, it is acceptable to ask them what tasks the dog does for them, what they need to make the visit a success, and if they are comfortable with a proposed treatment plan. The AVMA (98) also provides recommendations for working with clients with allergies or who are immunocompromised.

To qualify as an Emotional Support Animal (ESA) in the legal sense—for example, in order to be granted access and be exempt from additional fees, as covered under the U.S. Fair Housing Act (FHA) and the Air Carrier Access Act (ACAA)—the owner must possess a letter from a licensed human medical professional stating the animal's necessity for supporting the client's health. They may be required to provide this letter as proof of the animal's status. If asked by a client to provide a letter in support of the animal serving as an ESA, the veterinarian should decline; this letter needs to be written by a human medical professional familiar with the client's medical history (31). Because these animals are not task trained, they may look exactly like pets not serving as ESAs, and thus there is a high potential for fraudulent claims (11). The lack of training and socialization provided to some ESAs can result in the animal behaving inappropriately (for example, when the animal is aggressive, presents a risk to others in the facility, or is not under the handler's control). In these cases, staff may ask the client to leave the facility with the animal (11). The veterinarian should assist clients with ESAs in selecting an animal with a temperament suitable to that role, and should ensure on an ongoing basis that working ESAs are physically healthy and behaviorally appropriate for being taken out in public (31). This would include assessing whether the animal is displaying signs of stress or aggressive behavior when in locations where he/she may be taken in their role as an ESA (such as out in public, or in unfamiliar locations such as airplanes) (31).

In all cases, however, the veterinarian's mission is to provide care for the animal, and to make life better for both the animal and its human family (42). ESAs can provide essential support for many individuals with emotional, psychiatric, and other disabilities, and thus clinicians should consider how treatments or hospitalization, etc., will impact the ESA's ability to perform this function for the client.

## Conclusions

This review summarizes current recommendations for veterinarians working with clients who have disabilities and/or in cases when the patient is an assistance dog. Common themes emerge from the body of literature available on this topic:

- Clients with disabilities, and their pets and/or assistance animals, should receive the same quality of care as clients without disabilities.
- Providing this care may require flexibility, accommodations or alternate approaches, and team training.
- Extra time may be required in orienting and assisting clients with disabilities, to ensure that their needs are met and the veterinary visit is a success for both client and animal.
- Effective communication is paramount, and veterinarians should implement steps needed to best communicate with clients with specific disabilities.
- Veterinarians should ensure that the client understands and is willing and able to follow treatment recommendations.
- When in doubt about what the client needs to make his/her visit a success, staff should ask the client directly.
- When the patient is an assistance dog, the veterinarian should consider what impacts treatment will have on the dog's ability to function in its role for the client.
- Veterinarians should actively monitor physical and behavioral wellness of assistance animals, work regularly with persons responsible for their care, and educate owners of these animals about their physical and behavioral needs.
- Dogs living in kennels may be particularly at risk of behavioral problems, and veterinarians should be familiar with the signs of chronic stress in dogs.

## Author Contributions

EG conducted the literature review and wrote the manuscript. LH provided the original concept, contributed to the literature review, and commented on manuscript drafts.

### Conflict of Interest Statement

The authors declare that the research was conducted in the absence of any commercial or financial relationships that could be construed as a potential conflict of interest.

## Footnotes

^1^The ADA defines a “service animal” as “any dog that is individually trained to do work or perform tasks for the benefit of an individual with a disability, including a physical, sensory, psychiatric, intellectual, or other mental disability” (ADA 1990, Section 35.136). This definition is very similar to the more recent use of the term “assistance animal” used by ADI and others, under which definition “service animal” usually refers to assistance animals trained for work with people with disabilities other than those related to vision or hearing (see text for more on the ADI categories).

^2^Definitions and examples of invisible disabilities can be found at Disabled World. (2018-10-03) Invisible Disabilities: List and General Information. Available online https://www.disabled-world.com/disability/types/invisible/ (accessed October 2018).

## Abbreviations

AAI: Animal-assisted Interventions
AAT: Animal-assisted Therapy
ACAA: U.S. Air Carrier Access Act (1986)
ADA: Americans with Disabilities Act (1990)
ADHD: attention deficit hyperactivity disorder
ADI: Assistance Dogs International
AVMA: American Veterinary Medical Association
BCS: body condition score
DOT: U.S. Department of Transportation
ESA: emotional support animal
FHA: U.S. Fair Housing Act (Title VIII of the Civil Rights Act of 1968)
HUD: U.S. Department of Housing and Urban Development
PTSD: post-traumatic stress disorder
RP: responsible person (i.e. the individual responsible for animal's welfare)
TTY: teletypewriter (used in phone relay service).
